# Hybrid MambaVision and Transformer-Based Architecture for 3D Lane Detection

**DOI:** 10.3390/s25185729

**Published:** 2025-09-14

**Authors:** Raul-Mihai Cap, Călin-Adrian Popa

**Affiliations:** Department of Computers and Information Technology, Politehnica University of Timișoara, 300223 Timișoara, Romania; raul.cap@student.upt.ro

**Keywords:** 3D lane detection, Mamba vision, transformer networks, deformable attention, autonomous driving

## Abstract

Lane detection is an essential task in the field of computer vision and autonomous driving. This involves identifying and locating road markings on the road surface. This capability not only helps drivers keep the vehicle in the correct lane, but also provides critical data for advanced driver assistance systems and autonomous vehicles. Traditional lane detection models work mainly on the 2D image plane and achieve remarkable results. However, these models often assume a flat-world scenario, which does not correspond to real-world conditions, where roads have elevation variations and road markings may be curved. Our approach solves this challenge by focusing on 3D lane detection without relying on the inverse perspective mapping technique. Instead, we introduce a new framework using the MambaVision-S-1K backbone, which combines Mamba-based processing with Transformer capabilities to capture both local detail and global contexts from monocular images. This hybrid approach allows accurate modeling of lane geometry in three dimensions, even in the presence of elevation variations. By replacing the traditional convolutional neural network backbone with MambaVision, our proposed model significantly improves the capability of 3D lane detection systems. Our method achieved state-of-the-art performance on the ONCE-3DLanes dataset, thus demonstrating its superiority in accurately capturing lane curvature and elevation variations. These results highlight the potential of integrating advanced backbones based on Vision Transformers in the field of autonomous driving for more robust and reliable lane detection. The code will be available online.

## 1. Introduction

Detecting and pinpointing lanes on roads is an important task for computer vision and self-driving vehicles. Lane detection systems can help drivers stay in their lane and provide crucial information for advanced driver assistance systems and autonomous vehicles. Traditional lane detection methods use image processing techniques to recognize lane markings in camera images. Recently, deep learning has led to the creation of more precise and durable lane detection models based on convolutional neural networks (CNNs) and Transformer models. However, lane detection remains difficult due to varying road conditions, lighting conditions, camera perspectives, and the need for real-time performance.

There are two main approaches to lane detection: 2D and 3D models. Two-dimensional methods use CNNs such as ResNet [1], EfficientNet [2], and MobileNet [3,4] to detect lane markings in monocular images. These models are fast and effective in ideal conditions, but may struggle with poor weather, lighting, or degraded lane markings. In contrast, 3D models estimate the position and orientation of lane markings in 3D space, offering improved accuracy and robustness. Although most 3D models use inverse perspective mapping (IPM) [5] to create a bird’s-eye view (BEV), not all rely on this method. However, 3D models are generally more computationally intensive and depend on precise transformations.

Previous state-of-the-art methods hypothesized wrongly the construction of the BEV image, assuming that the lines have no height. This made the obtained image not accurate because they started from the premise that the world is flat. The authors of those works tried to determine the coordinates in the 3D space of the traffic lanes depending on the curvature of the lane lines. It is understandable that it was very difficult, nearly impossible, to generate a more accurate top-down view image because, at that time, there were no datasets in which the exact coordinates of the traffic lanes could be mapped in 3D space. At that time, there existed only synthetic datasets such as Apollo [6], which were obtained from various game engines.

Developing a 3D lane detection dataset poses a significant challenge, primarily due to the requirement of precise and comprehensive ground truth annotations. Typically, this process involves manually labeling lane markings in a 3D space, which can be time-consuming and requires specialized equipment, such as LiDAR sensors.

Another challenge relates to generating a diverse and representative set of scenes that accurately capture a wide range of real-world scenarios, including various road types, weather, and lighting conditions. This undertaking requires careful planning, execution, and access to appropriate data collection sites.

Fortunately, we now have at our disposal three large datasets that contain a multitude of driving scenarios from real situations, whose annotations are very well detailed. The datasets to which we refer are OpenLane [7], OpenLaneV2 [8], and ONCE-3DLanes [9]. One of the things that differentiates these datasets is the evaluation metrics. A well-constructed dataset plays a crucial role for lane detection tasks because, when annotations are inconsistent, training becomes noisy and slow, and the resulting model struggles to generalize. Imperfect ground truths such as missed or misaligned lane markings can degrade the performance of deep neural networks, which is supported by recent results from the PSSCL framework [10]. Furthermore, careful selection of cleaner data during training significantly improves the robustness to such label noise.

Certainly, there are several methods that can work alongside those mentioned in order to obtain better results. These methods involve the inclusion of several sensors, such as RADAR or LiDAR, in contemplation of better understanding the surrounding environment by generating point clouds or depth maps. The main disadvantage of those methods is that, as we add more sensors, the computational cost increases drastically and it becomes more and more difficult and expensive to be used in real life.

Transformers are increasingly being used in computer vision applications such as image classification, object detection, and semantic segmentation [11,12], as they are capable of capturing long-range dependencies and contextual information in the input data. Unlike CNNs, which process input data sequentially and extract features using local receptive fields, Transformers utilize a self-attention mechanism to compute global interactions between all input elements, thereby enabling them to capture both local and global contextual information. However, their computational requirements grow exponentially with sequence length, creating challenges for training and deployment.

For computer vision tasks, Transformer-based models such as Vision Transformers (ViT) [13], DETR [14], and SETR [15] have demonstrated state-of-the-art performance on challenging datasets such as ImageNet [16], COCO [17], and Cityscapes [18]. Transformers have several advantages over CNNs, including the ability to model long-range dependencies and context more effectively, less dependence on predefined architectures, and the ability to handle variable-sized inputs. However, with the recent introduction of Mamba Vision models, even better results have been achieved, surpassing the performance of ViTs in many computer vision tasks by combining the strengths of state-space models and Transformers for enhanced accuracy and efficiency.

The main objective of our research is to integrate the Transformer [19] and Mamba Vision [20] architectures into current state-of-the-art models and replace the classic convolutional neural networks in order to achieve better results.

The main contributions of the paper are as follows:

- We propose a simple hybrid backbone that mixes MambaVision-S-1K CNN blocks with a Transformer, so the model learns both fine details and global context.
- Our approach works directly on front-view images and skips any IPM or bird’s-eye view step.
- We fine-tune and optimize the whole model so it can run well on systems which do not require the latest and best configuration.
- We achieve state-of-the-art results on ONCE-3DLanes and also very good results on all subsets of the Apollo 3D Lane Detection benchmark.

The code will be available at https://github.com/raul-cap/mamba-3d-lane-detection (accessed on 29 July 2025).

## 2. Related Work

A study on 3D-LaneNet [21] has applied two new concepts: an intra-network inverse perspective mapping that provides a dual representation information flow for both a regular image captured by the camera and its top-down view, and an anchor per column representation, which makes the lane detection task more like an object detection problem, replacing the common methods such as outlier rejection and clustering. This is the first work to make use of the top-view representation of the standard image to determine the lane in 3D space.

Being the first work to approach this topic, 3D-LaneNet inspired most of the works that followed, as well as the work entitled “Gen-LaneNet: A Generalized and Scalable Approach for 3D Lane Detection” [6]. With this paper, the authors noticed and tried to remove the drawbacks of 3D-LaneNet. One of the drawbacks is that the bird’s-eye projection does not align with the image feature resulting from IPM in the presence of a non-zero slope. Ref. [21] uses an unsuitable coordinate frame in its anchor representation, causing ground truth lanes to be misaligned with visual features. On the other hand, the authors proposed the so-called “virtual top view”, which aligns with the IPM image. Ref. [22] is also an extension of the first state-of-the-art methods [6,21]. The authors have introduced a new attention mechanism called Dual Attention. Its purpose is to help the model work better and improve accuracy in more complicated driving situations and scenarios. Besides this attention module, another change compared to the previous models is the use of the linear-interpolation loss function, which is used to locate the 3D lane markings with greater precision.

Although other previously presented works all had 3D-LaneNet as their starting point and each work represented an improvement of the basic one, in CLGo [23], the authors applied a different strategy. Among the only similarities to the other works is that its architecture has two stages. However, the first stage is no longer used for image segmentation, but instead for finding the best camera pose and polynomial parameters using geometric constraints. In this case, finding the best camera pose is equivalent to finding the camera height and the pitch angle of the camera. This is performed by using a Transformer encoder–decoder.

The backbone of this work replaces the classic backbones that used convolutional neural networks with one based on Transformers, which are mainly used to enhance image features. The convolution is only used to extract the convolutional features that will be inputs for the Transformer encoder (TRE). By decoding the characteristics using the Transformer decoder (TRD), the camera pose is obtained. The authors added a lane branch to interpret 3D lanes to aid in camera pose learning. First, the 3D lanes are obtained, and then they are projected into the 2D plane by conducting a homographic transformation. Thus, the model learns the best camera pose by comparing the results to the ground truths in both 3D and 2D planes.

In [7], the authors proposed a method entitled PersFormer, which is an end-to-end monocular 3D lane detector model that can simultaneously detect 2D and 3D lanes. Like the latest state-of-the-art models, the method is based on the Transformer architecture. However, the Transformer is not used as the backbone for feature extraction, which is the first part of this network. The second part of the model is the Perspective Transformer, which is relatively complex, and also the inspiration for the name of the model. The general idea behind the Perspective Transformer is to generate an accurate BEV of the input image by taking into consideration the front view features generated by the ResNet [1] backbone, the coordinates of the transformation matrix from IPM, and the camera intrinsic and extrinsic parameters, such as camera height, and the pitch angle of the camera. The authors used a Transformer to attend local context and aggregate global features to produce a solid representation in BEV rather than just projecting the one-to-one feature correspondence from front view to BEV. In addition to the proposed model, the authors highlighted one of the existing problems in the 3D lane detection task, namely, the lack of data for the learning process. They addressed this problem by introducing a new large-scale dataset called OpenLane.

With the same purpose as [7], previously presented, ONCE-3DLanes [9] addresses the problem of insufficient data for 3D lane detection and introduces a new real-world autonomous driving dataset to encourage development on this subject. In addition to this, the authors also proposed a model for 3D lane detection called SALAD that does not require human-made anchors and also does not require transformation to BEV or top-view.

In [24], a lane-aware query generator is introduced, which adapts to lane-specific characteristics and improves detection accuracy by jointly leveraging lane-level and point-level embeddings. Furthermore, LATR uses a Dynamic 3D Ground Positional Embedding that iteratively refines a 3D ground plane to better align with the actual road geometry. Thus, it addresses the limitations of traditional assumptions related to a fixed 3D space. Using these algorithms, the method achieved state-of-the-art results on the ONCE-3DLanes dataset and very impressive results on other datasets without using BEV image generation.

The state-space model (SSM) introduced by Mamba [25] addresses ViT problems by providing linear-time complexity. Its results match or even exceed the performance of Transformers in natural language processing tasks. Mamba’s key innovation lies in the efficient handling of long sequences, making it a scalable solution compared to traditional Transformers.

Vision Mamba [26] further improves upon this by incorporating bidirectional SSMs to mitigate the weaknesses of Transformers in capturing global context and spatial relationships. However, the added complexity of processing sequences in bidirectional SSMs can lead to challenges such as increased latency, overfitting, and uncertainty in accuracy improvement. Despite these challenges, ViTs and CNNs often outperform Mamba-based models in visual tasks because of their efficiency and reliability.

Recently, hybrid models that combine Mamba and Transformer architectures have gained considerable attention. Ref. [20] introduced MambaVision, a novel hybrid model designed for computer vision tasks that combines the strengths of both Mamba and Transformers. It employs a hierarchical structure with multi-resolution CNN-based residual blocks to swiftly extract features across different resolutions.

Related to our design choices, we also consider advances in detection architectures beyond lane detection. Ref. [27] shows how combining multiple feature streams can remain robust even when one modality, for example, thermal or RGB, is noisy or unreliable. This idea is similar to our goal of making geometric cues robust against imperfect or uncertain signals. In another line of work, ref. [14] replaces dense attention with sparse, reference-point-based attention, which improves greatly the efficiency and accuracy in detection. This mechanism directly inspires the use of deformable attention in the decoder in our work.

## 3. Method Overview

Motivated by the success of the latest state-of-the-art LATR [24] model, we propose a 3D lane detection model with Dual Transformer. In this section, we will present the overall architecture and implementation details.

The objective of the proposed solution is to obtain the coordinates of the traffic lanes in a three-dimensional space, starting from an image that is captured by a camera mounted on the front of the vehicle. Because we do not generate a BEV image for the lane detection, we no longer require precise calibration of the well-defined intrinsic and extrinsic parameters of the camera, such as the height of the camera, the focal length, or the pitch angle, as we previously did in [6,21]. Concretely, we still use a simple pinhole projection to map 3D points to the image, but we let the ground plane be adjusted during training with two residuals corresponding to pitch $\left(\Delta \theta_{x}\right)$ and height (Δh), which are supervised in the lane pixels by $L_{\mathrm{plane}}$. On ONCE-3DLanes, the camera extrinsics are unavailable. So, we follow previous works [7,24] and use approximate camera settings, and the learned plane compensates for the resulting mismatch. Thus, we can use this method to approximate the camera extrinsics on every dataset.

**Figure 1:**
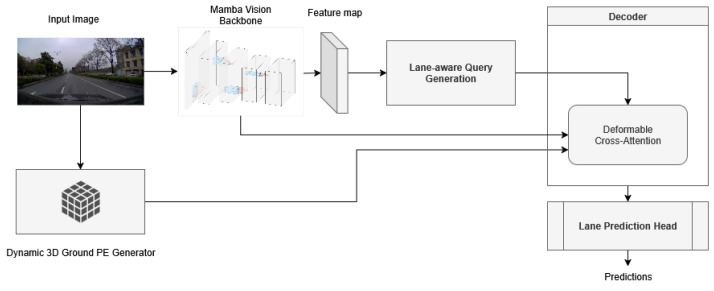
Proposed method architecture. The model begins by extracting a feature map *X* using the MambaVision backbone, followed by generating lane-aware queries *Q* that refine the feature map $X^{\prime }$ through a deformable attention mechanism. Next, the Dynamic 3D Ground Positional Embedding introduces 3D context, updating the feature map to $X^{\prime \prime }$, after which the dynamic plane is iteratively updated to align with real-world geometry using a transformation matrix, and, finally, the prediction head processes the refined feature map to produce accurate 3D lane predictions.

The architecture of our proposed model is shown in Figure 1. The process begins by extracting feature maps from the input image using the MambaVision backbone, which was chosen for its ability to capture both fine-grained details and broader contextual features effectively. After this important step, the incoming process is inspired and similar to LATR. Lane-aware queries are generated, where the number of queries corresponds to the number of lanes, and the number of points represents each lane. These queries are then designed to interact with the extracted feature maps using a deformable attention mechanism, which allows the model to dynamically focus on the most relevant parts of the image.

To further enhance the model’s understanding of the scene, we employ a Dynamic 3D Ground Positional Embedding Generator. This component integrates three-dimensional spatial information into the two-dimensional features which ensure that the model accurately reflects lane geometry without relying on static 3D structures. Finally, a prediction head processes the enhanced queries and leads to the generation of the final lane predictions. Each component of this architecture will be explained in more detail in the following sections.

### 3.1. Backbone

MambaVision serves as the feature extraction backbone for the object detection and segmentation models. In these frameworks, the backbone network is responsible for extracting rich feature maps from the input image, which are then used by subsequent layers to detect objects and generate segmentation masks. The hierarchical architecture of MambaVision, which includes both CNN-based residual blocks and Transformer blocks, makes it highly effective for this role.

CNN layers in MambaVision, specifically in Stages 1 and 2, quickly process high-resolution features. Given an input image $X\in R^{H\times W\times 3}$, where *H* and *W* are the height and width of the input image, which is an RGB image with three channels, the number of channels is denoted with *C*. These layers extract feature maps $F\in R^{\frac{H}{2}\times \frac{W}{2}\times C}$ through a series of convolutions and pooling operations:

F=CNN(X),

where CNN(·) denotes the convolutional operations, including residual connections, which are formulated as

$$F_{\mathrm{res}}=\mathrm{Conv}\left(\sigma \left(\mathrm{BN}\left(F\right)\right)\right)+F.$$

Here, σ represents the activation function, typically ReLU, and BN denotes batch normalization. These residual connections help maintain spatial details crucial for accurately locating and identifying objects by capturing both low-level and high-level features.

In Stages 3 and 4, MambaVision employs a combination of MambaVision Mixer blocks and Transformer blocks. The MambaVision Mixer blocks utilize Structured SSMs to capture both short- and long-range dependencies. The SSM in MambaVision can be expressed using the following state-space equations:

$$h^{\prime }\left(t\right)=Ah\left(t\right)+Bx\left(t\right),\;y\left(t\right)=Ch\left(t\right),$$

where $h\left(t\right)\in R^{M}$ is the hidden state, x(t)∈R is the input, and y(t)∈R is the output. The parameters $A\in R^{M\times M}$, $B\in R^{1\times M}$, and $C\in R^{1\times M}$ are learnable. These parameters are discretized for computational efficiency. This allows the MambaVision Mixer to process sequences effectively and capture complex dependencies.

The output of the MambaVision Mixer block can be represented as

$$F_{\mathrm{mixer}}=\mathrm{Mixer}\left(F_{\mathrm{low}}\right),$$

where $F_{\mathrm{low}}\in R^{\frac{H}{4}\times \frac{W}{4}\times C^{\prime }}$ are the lower-resolution feature maps. The Mixer block combines convolutional operations with SSMs, capturing both local and global context.

The Transformer blocks in Stages 3 and 4 further enhance the model’s ability to maintain global context. Using the multi-head self-attention mechanism, the Transformer blocks process the feature maps $F_{\mathrm{mixer}}$ as

$$\mathrm{Attention}\left(Q,K,V\right)=\mathrm{softmax}\left(\frac{QK^{T}}{\sqrt{d_{k}}}\right)V,$$

where Q, K, and V are the query, key, and value matrices derived from $F_{\mathrm{mixer}}$, and $d_{k}$ is the dimensionality of the key vectors. This self-attention mechanism allows the model to capture dependencies across the entire image to ensure a comprehensive understanding of object relationships and spatial configurations.

The combined output from the MambaVision Mixer and Transformer blocks provides enriched feature maps that are well-suited for generating accurate segmentation masks and object detections:

$$F_{\mathrm{out}}=\mathrm{Transformer}\left(F_{\mathrm{mixer}}\right),$$

where Transformer(·) denotes the transformation applied by the Transformer block. This hierarchical combination of CNNs, SSMs, and Transformers within MambaVision makes it a powerful backbone for object detection and segmentation tasks, which balances both computational efficiency and accuracy.

The LATR model begins by processing the input image *I* using the MambaVision-S-1K backbone to extract a feature map *X*. The feature map captures the necessary visual details from the front view of the driving scene and provides a rich set of features for further processing:

$$X\in R^{C\times H\times W}=\mathrm{MambaVision}\left(I\right).$$

Here, *C* is the number of channels, *H* is the height, and *W* is the width of the feature map.

### 3.2. Lane-Aware Query Generation

After extracting the feature map, the model generates lane-aware queries *Q* using the lane-aware query generator. These queries are tailored to each detected lane and incorporate both lane- and point-level embeddings. The lane-aware queries *Q* are represented as

$$Q=Q_{\mathrm{lane}}⊕Q_{\mathrm{point}},$$

where $Q_{\mathrm{lane}}$ captures the overall lane structure and is computed using instance activation maps (IAMs) derived from the feature map *X*, and $Q_{\mathrm{point}}$ captures detailed information at specific points along the lane, leveraging the learnable weights corresponding to the predefined longitudinal coordinates.

### 3.3. Interaction via Deformable Attention

The lane-aware queries *Q* then interact with the feature map *X* through a deformable attention mechanism. This mechanism allows the model to selectively focus on relevant regions in the image by dynamically adjusting its attention based on the queries:

$$X^{\prime }=\mathrm{DeformAttn}\left(Q,X\right),$$

where $X^{\prime }$ is the refined feature map after the deformable attention operation, which uses the lane-aware queries to capture relevant features for lane detection.

### 3.4. Dynamic 3D Ground Positional Embedding (PE) Generation

To integrate three-dimensional context, the model employs a Dynamic 3D Ground Positional Embedding Generator. This component enhances the feature map $X^{\prime }$ with 3D spatial information by projecting a hypothetical 3D ground plane into the 2D feature space:

$$d\cdot {\left[u,v,1\right]}^{T}=T\cdot {\left[x,y,z,1\right]}^{T},$$

where (u,v) are the 2D coordinates on the feature map $X^{\prime }$ (in pixels), d>0 is the homogeneous scale factor of the projection, ${\left[x,y,z,1\right]}^{⊤}$ is a 3D point on the ground plane, and *T* is the pinhole camera projection matrix.

A 3D ground plane *P* is initialized and iteratively refined using a transformation matrix *D* to align with the real-world ground. This embedding step enhances the feature map $X^{\prime \prime }$, which allows the model to accurately represent the lane geometry:

$$X^{\prime \prime }=\mathrm{PEGenerator}\left(X^{\prime }\right).$$

During training, the ground plane is adjusted step by step by adjusting the pitch and height. We train it together with the lane losses using a simple plane alignment loss (Section 3.7). This learned, adjustable plane works better than a fixed plane or a fixed frustum.

### 3.5. Iterative Plane Update

We describe the transformation matrix *D* using the pitch angle $\left(\Delta \theta_{x}\right)$ and the vertical shift (Δh). With these, we construct the matrix:

$$D=\left[\begin{array}{cccc} 1 & 0 & 0 & 0\\ 0 & \cos\Delta \theta_{x} & -\sin\Delta \theta_{x} & 0\\ 0 & \sin\Delta \theta_{x} & \cos\Delta \theta_{x} & \Delta \;h\\ 0 & 0 & 0 & 1 \end{array}\right].$$

*D* is used to update the 3D ground plane, adjusting its position to better fit the ground truth road surface. In the decoder layer *t*, the plane grid from the previous step, $P_{t-1}$, is updated by multiplying it by this matrix:

$$P=D\cdot P_{t-1}.$$

The update is based on the residuals calculated between the predicted 3D ground plane and the actual 3D lane annotations.

### 3.6. Prediction Head for Final Lane Predictions

Finally, the enhanced feature map $X^{\prime \prime }$ is fed into a prediction head that uses an MLP to output the final 3D lane predictions. This head estimates the 3D coordinates of the lane points and their visibility:

[Δx,Δz,v]=MLP(Q).

This final step produces the predicted 3D positions and classifications of the lanes, providing a comprehensive 3D lane model. This workflow avoids the use of traditional 3D surrogates like IPM and leverages Transformer-based attention mechanisms for efficient and accurate 3D lane detection.

### 3.7. Loss Function

The loss function is computed the same as in [24] and combines several components to ensure accurate 3D lane detection, focusing on regression, visibility, and classification.

The primary loss for 3D lane prediction $L_{\mathrm{lane}}$ consists of

$$L_{\mathrm{lane}}=w_{x}L_{x}+w_{z}L_{z}+w_{v}L_{v}+w_{c}L_{c},$$

where $L_{x}$ and $L_{z}$ are regression losses for the *x*- and *z*-coordinates of the lane points, $L_{v}$ is the visibility loss, using binary cross-entropy, and $L_{c}$ is the classification loss, handled by focal loss to manage class imbalance.

The overall loss function is a weighted combination of 3D lane prediction, segmentation, and 3D ground plane alignment losses:

$$L=w_{s}L_{\mathrm{seg}}+w_{p}L_{\mathrm{plane}}+w_{l}L_{\mathrm{lane}},$$

where $L_{\mathrm{seg}}$ trains a simple lane versus background mask obtained by drawing the ground-truth lane lines on the image and $L_{\mathrm{plane}}$ makes the learned ground plane, composed of pitch and height, to match the 3D lane annotations at labeled pixels.

We supervise the ground plane with a simple alignment loss computed only at lane pixels:

$$L_{\mathrm{plane}}=\sum_{(u,v)\in L}{∥M_{p}\left(:,u,v\right)-M_{\ell }\left(:,u,v\right)∥}_{2},$$

where $M_{p}\in R^{3\times H\times W}$ is the 3D canvas induced by the current ground plane and $M_{\ell }$ stores the metric 3D of the annotated lane points. Training is end-to-end, so this loss is optimized together with the lane losses, and gradients pass through the plane update, so the plane aligns to the road surface over time.

The values we have used for the base weights are the following: $w_{s}\;=\;5.0$, $w_{x}\;=\;2.0$, $w_{z}\;=\;10.0$, $w_{c}=10.0$, and all others =1.0. We find that they are stable across both datasets.

This structure ensures that the model optimizes not only for accurate lane positioning but also for visibility and correct ground plane alignment.

## 4. Experiments

In this section, we present the training details alongside the results of the proposed model on the ONCE-3DLanes (https://once-3dlanes.github.io/3dlanes/, accessed on 29 July 2025) and Apollo 3D Lane Detection (https://developer.apollo.auto/synthetic.html, accessed on 29 July 2025) datasets.

### 4.1. Training Details

In our experiments, we set the batch size to 2 and used three workers during training. The number of categories for classification is 2, one representing the lane and the other the background, and we defined a positive threshold of 0.3 for classification. The labels corresponding to the lane and background created labels by drawing the ground-truth lane lines on the image and making them a bit thicker, 3 pixels at 1/4 of the resolution of the feature map. The pixels on these thick lines are labeled ’lane’ and all other pixels are ’background’. This auxiliary head is used only during training and it is dropped at inference.

Our model follows an encoder–decoder architecture based on [24]. We resize the input images to a resolution of 720 × 960 and they are then fed to the backbone. The encoder is initialized with the MambaVision-S-1K pretrained model, and we employ a Feature Pyramid Network (FPN) as the neck, with input channels [192, 384, 768] and output dimension of 192. Those input channels correspond to the feature map channels extracted from the last three stages of the MambaVision backbone, which represent different levels of spatial detail. The FPN outputs four feature maps and each one is passed through additional convolutions with the largest scale aggregated as input for the decoder.

For the decoder, we apply deformable attention with four heads, eight sample points, and 192-dimensional embeddings. A six-layer Transformer decoder is used for object detection and segmentation, with 12 queries and 20 anchor points per query distributed across the *y* axis.

All models are trained using the AdamW optimizer with a learning rate of $2\times 10^{-4}$ and a weight decay of 0.01. We set the learning rate multiplier for sampling_offsets to 0.1.

The proposed method was trained and evaluated on a system that has the following specifications: Intel 4790K CPU (Intel, Santa Clara, CA, USA), one NVIDIA RTX 3060 Ti GPU (NVIDIA, Santa Clara, CA, USA), 16GB DDR3 RAM, and 1TB SSD storage. We strongly believe that slightly better results can be achieved by fine-tuning the hyper-parameters, but due to our system limitation, these were the best results we could achieve.

### 4.2. Evaluation Metrics

The evaluation metrics used to evaluate the performance of the proposed model on the ONCE-3DLanes dataset are the F1 score, precision, recall, and CD error. Precision represents the ratio between all correctly identified positive instances and all instances that were predicted as positive. Recall refers to the ratio of true positives out of all the actual positive instances in the dataset. The F1 score represents the harmonic mean between precision and recall. Those three metrics can be mathematically expressed as

$$\mathrm{Precision}=\frac{TP}{TP+FP},\;\mathrm{Recall}=\frac{TP}{TP+FN},\;F1=2\;\frac{\mathrm{Precision}\times \mathrm{Recall}}{\mathrm{Precision}+\mathrm{Recall}},$$

where TP is the number of true positives, FP the number of false positives, and FN the number of false negatives.

The CD error represents the Chamfer distance between the predicted lane line and the ground truth.

The Apollo dataset uses the F1 score and, besides it, errors that are computed using the Euclidean distance and measured in meters. It compares the matched lanes for the near range (0–40 m) and the far range (40–100 m).

### 4.3. Results on ONCE-3DLanes

The ONCE-3DLanes [9] dataset is a collection of annotated data for autonomous driving, which includes lane layout information in 3D space. To create this dataset, a pipeline was developed that can automatically generate precise 3D lane location data from 2D annotations. This is achieved by utilizing the explicit relationship between point clouds and image pixels, having over 211,000 road scenes, and resulting in a high-quality dataset. The model was trained for 20 epochs, with the best results being achieved in the 11th epoch.

The results obtained by our model on the ONCE-3DLanes dataset, as shown in Table 1, indicate a significant improvement over current state-of-the-art methods across all key metrics. Specifically, our model achieved an F1 score of 82.39%, outperforming the previous highest score of 80.59% achieved by LATR [24]. This indicates a superior balance between precision and recall in detecting lane markings.

### 4.4. Results on Apollo 3D Lane Detection

The Apollo 3D Lane Detection dataset, introduced in [6], is an extension of the original Apollo Synthetic Dataset. The dataset was built using the Unity game engine to encourage research and development in the field of autonomous driving. The main reason behind it was the lack of labeled real-world data. The Apollo 3D Lane Detection dataset contains a total of 6000 samples from the virtual highway map, 1500 samples from the urban map, and 3000 samples from the residential area, along with the corresponding depth map, semantic segmentation map, and 3D lane line information.

The dataset is split into three sets to evaluate the algorithms. The “balanced scenes” set follows a standard five-fold split for unbiased data training and testing. The “rarely observed scenes” set uses a subset of testing data from a complex urban map to evaluate generalization capabilities. The “scenes with visual variations” set evaluates methods under illumination changes, such as 3D examples from before dawn.

The performance of our model on the Apollo 3D Lane Detection dataset, particularly in the balanced scenes category, Table 2, highlights its strength compared to current state-of-the-art methods. Our model achieved an F1 score of **97.0%**, which is second after the best of **97.4%** achieved by [30]. This performance in F1 score indicates that our model strikes an excellent balance between precision and recall, outperforming models like [24,28], which also performed well but fell slightly short in this aspect.

For the rarely observed and visual variant scenes, as shown in Table 3 and Table 4, our model delivers solid performance. In the rarely observed scenes, while [32] achieves the highest F1 score, our model remains competitive with an F1 score of 95.1%, positioning it closely to other top-performing methods. Similarly, in the visual variant scenes, our model achieves an F1 score of 94.5%, indicating reliable performance across diverse environmental and visual conditions.

Our backbone, MambaVision-S-1K, is composed from the following hybrid blocks: Conv, Mamba, Transformer. The Mamba and the self-attention blocks from the Transformer have the ability to capture and model long-term relationships, but weaker inductive biases than CNNs. Thus, they rely more on the amount and diversity of data to learn these relationships correctly. In [20], it was observed that, when trained only on ImageNet-1K, which has around 1.2 M images, MambaVision-S performs comparably to ResNet, but really gains advantage on larger sets such as ImageNet-22K. As the Apollo subsets have much fewer images than ONCE-3DLanes, it was expected that our configuration would not perform as well on this dataset as on ONCE-3DLanes. Although there is room for improvement in these more challenging scenarios, our model still shows good overall performance.

### 4.5. Runtime and Complexity Comparison

At our evaluation resolution, 720 × 960, our MambaVision-S-based model counts 62.6 M parameters and 175.5 GFLOPs (FLOPs $\times 10^{9}$), in comparison to LATR, which uses the ResNet-50 backbone and has 46.8 M parameters and 127.9 GFLOPs. Those results were not presented in [24], but we used their official GitHub repository to download a pretrained model and calculate the complexity under the same input. Although this may seem like an increase in complexity, our model remains substantially more efficient than PersFormer, which is the baseline model for LATR in this line of work, despite being moderately heavier than [24]. The authors of [7] did not publish the complexity of the model, but thanks to GroupLane’s work [33], we were able to obtain them. PersFormer working with EfficientNet-B7 [2] is much heavier, having 572.4 GFLOPs, which is ∼3.26 times more than our computational cost.

We ran the inference on our system, which uses NVIDIA RTX 3060 Ti GPU (NVIDIA, Santa Clara, CA, USA), with batch size 1 and excluded the first five warmup iterations. We measure 14.44 FPS for our model versus 15.84 FPS for LATR under the same configuration. In terms of latency, it is ∼69.3 ms per frame for ours and ∼63.1 ms for LATR, which represents a difference of ∼6.1 ms (∼8.8% in FPS). In practice, this is a modest difference on our system, and both models operate in essentially almost the same real-time regime at this resolution.

## 5. Conclusions

In conclusion, we have proposed an innovative 3D lane detection model that uses the MambaVision backbone integrated with Transformer-based attention mechanisms. This approach significantly improves lane detection performance in 3D space and addresses the challenges posed by non-flat surfaces and variable road conditions. Using deformable attention and lane-aware queries, the model dynamically focuses on relevant regions of the image, resulting in enhanced accuracy for lane detection tasks. Our method achieved state-of-the-art results on the ONCE-3DLanes dataset and competed closely with other methods on the Apollo 3D Lane Detection dataset, demonstrating its superiority in terms of precision, recall, and F1 score. These results highlight the potential of Transformer-enhanced backbones for robust 3D lane detection in autonomous driving applications.

## Figures and Tables

**Table 1:** Evaluation results on ONCE-3DLanes. Best values are highlighted in bold.

Model	F1 (%)	Precis. (%)	Recall (%)	CD Err. (m)
3D-LaneNet [21]	44.73	61.46	35.16	0.127
Gen-LaneNet [6]	45.59	63.95	35.42	0.121
SALAD [9]	64.07	75.90	55.42	0.098
PersFormer [7]	74.33	80.30	69.18	0.074
Anchor3DLane [28]	74.87	80.85	69.71	0.060
WS-3D-Lane [29]	77.02	84.51	70.75	0.058
LATR [24]	80.59	**86.12**	75.73	**0.052**
**Ours**	**82.39**	85.09	**79.85**	0.055

**Table 2:** Evaluation results on Apollo 3D Lane Detection balanced scenes. Best values are highlighted in bold.

Model	F1 (%)	X Error Near (m)	X Error Far (m)	Z Error Near (m)	Z Error Far (m)
3D-LaneNet [21]	86.4	0.068	0.477	0.015	**0.202**
Gen-LaneNet [6]	88.1	0.061	0.469	0.012	0.214
CLGo [23]	91.9	0.061	0.361	0.029	0.250
PersFormer [7]	92.9	0.054	0.356	0.010	0.234
Anchor3DLane [28]	95.6	0.052	0.306	0.015	0.233
CurveFormer [31]	95.8	0.078	0.326	0.018	0.219
LATR [24]	96.8	0.022	0.253	**0.007**	**0.202**
BEV-LaneDet [32]	96.9	**0.016**	**0.242**	0.020	0.216
LaneCPP [30]	**97.4**	0.030	0.277	0.011	0.216
**Ours**	97.0	0.024	0.255	0.009	0.204

**Table 3:** Evaluation results on Apollo 3D Lane Detection rarely observed scenes. Best values are highlighted in bold.

Model	F1 (%)	X Error Near (m)	X Error Far (m)	Z Error Near (m)	Z Error Far (m)
3D-LaneNet [21]	72.0	0.166	0.855	0.039	**0.521**
Gen-LaneNet [6]	78.0	0.139	0.903	0.030	0.539
CLGo [23]	86.1	0.147	0.735	0.071	0.609
PersFormer [7]	87.5	0.107	0.782	0.024	0.602
Anchor3DLane [28]	94.4	0.094	0.693	0.027	0.579
CurveFormer [31]	95.6	0.182	0.737	0.039	0.561
LATR [24]	96.1	0.050	0.600	**0.015**	0.532
LaneCPP [30]	96.2	0.073	0.651	0.023	0.543
BEV-LaneDet [32]	**99.1**	**0.031**	**0.594**	0.040	0.556
**Ours**	95.1	0.076	0.626	0.022	0.536

**Table 4:** Evaluation results on Apollo 3D Lane Detection visual variant scenes. Best values are highlighted in bold.

Model	F1 (%)	X Error Near (m)	X Error Far (m)	Z Error Near (m)	Z Error Far (m)
3D-LaneNet [21]	72.5	0.115	0.601	0.032	0.230
Gen-LaneNet [6]	85.3	0.074	0.538	**0.015**	0.232
CLGo [23]	87.3	0.084	0.464	0.045	0.312
PersFormer [7]	89.6	0.074	0.430	**0.015**	0.266
LaneCPP [30]	90.4	0.054	0.327	0.020	**0.222**
CurveFormer [31]	90.8	0.125	0.410	0.028	0.254
Anchor3DLane [28]	91.4	0.068	0.367	0.020	0.232
LATR [24]	95.1	0.045	**0.315**	0.016	0.228
BEV-LaneDet [32]	**96.9**	**0.027**	0.320	0.031	0.256
**Ours**	94.5	0.067	0.337	0.018	0.231

## Data Availability

Data are contained within the article.
